# Argon Laser: A Modality of Treatment for Trichiasis

**Published:** 2007-03

**Authors:** Muawyah D. Al-Bdour, Maha I. Al-Till

**Affiliations:** *Ophthalmology Department Jordan University Hospital Amman, Jordan*

**Keywords:** argon laser, eyelash, Jordan, trichiasis

## Abstract

The purpose of this study is to evaluate the success rate and safety of argon laser photocoagulation as a modality of treatment for symptomatic trichiasis in a Middle Eastern country (Jordan). This simple descriptive study included 54 patients (68 lids) with symptomatic trichiasis. They were treated with argon laser and followed over 8 month period. After topical and infiltration anaesthesia, 30-40 shots of blue-argon laser were directed to the lash root to a depth of 2-3mm. Laser beam variables were: 50-100 μm spot size, 0.3 second duration and 0.50 Watt power. The maximum number of treated lashes per lid was five. Recurrence was defined as regrowth of one or more trichiatic lash. Up to two additional applications with the same laser parameters were done for recurrent trichiasis. The success rate after one treatment session was 61.1% and with up to a total of three sessions, it was 85.2%. The complication rate was 20.6% (14 lids) in the form of mild hypopigmentation in 8 lids and notching in 6 lids. The complication rate was higher among patients treated previously with cryotheray or lid surgery. Argon laser photocoagulation is a safe and effective office procedure for treatment of symptomatic trichiasis.

## INTRODUCTION

Trichiasis is a condition in which the lashes are abnormally directed posteriorly towards the surface of the eye. There has been a variety of treatment modalities used over the years for the management of symptomatic trichiasis including epilation, electrolysis, cryotherapy, and lid surgery. Mechanical epilation is a temporary measure, because most lashes regrow in 4 to 6 weeks. Electrolysis is a tedious procedure with recurrence rates as high as 50% (1). Cryotherapy is considered to be the most effective method of treatment with cure rates ranging from 70% to 90%. However, this procedure is associated with significant risk of complications including lid swelling, notching, skin depigmentation, reactivation of herpes zoster and other more serious sequelae (2-4).

In 1979, Berry (5) first published results of argon laser in the treatment of trichiasis. Since then argon laser has been used for the treatment of trichiasis with considerable advantages. Our prospective study was conducted at Jordan University Hospital between January 2005 and February 2006. The study aimed at evaluating the efficacy of argon laser photocoagulation as a modality of treatment for symptomatic trichiasis.

## MATERIALS AND METHODS

This simple descriptive study included 54 patients. Sixty eight lids were treated by argon laser to ablate symptomatic trichiatic lashes involving the upper eyelid, lower eyelid or both. The study protocol was reviewed and approved by Research and Ethics Committee at School of Medicine/University of Jordan. The procedure and chances of recurrence were explained to each patient after whom a consent form was signed by patients who agreed to be included in the study.

Prior to treatment, detailed history was taken for each patient. History of previous treatment (including mechanical epilation) for trichiatic lashes was recorded. Ophthalmic examination was performed. The aetiology of trichiasis was determined whenever possible. We used 0.4% Benoxinate hydrochloride eye drop for topical anaesthesia. The eyelid was anesthetized by subcutaneous injection of 0.5 ml of 2% lidocaine with (1:100,000) epinephrine in the region of trichiasis. Patient was asked to gaze away from the planned area of treatment. The eyelid was rotated slightly outward to align the root of misdirected lash with the laser beam. No protective lenses were used. Blue-green argon laser unit coupled with slit lamp was used. Laser beam variables were: 50-100 μm spot size, 0.3 second duration and 0.50 Watt power. The initial burn was focused at the base of the lash. It was sufficient to vaporize the cilium and to create a crater at the lid margin. The burns were applied deeper into the eyelid substance at the created crater. To ensure destruction of the hair follicle, treatment was applied to a depth of 2 to 3 mm with an average of 30-40 burns per eyelash. The maximum number of treated lashes per lid was five. Postoperatively, antibiotic-corticosteroid eye ointment was applied three times daily for one week. Every patient was followed up monthly during the first 4 months and thereafter bimonthly for a total of 8 months. Any patient who did not attend follow up clinics was not included in the study.

Recurrence was defined as regrowth of one or more trichiatic lash per lid. Up to 2 additional applications with the same parameters were done for recurrent trichiasis. Any complication like hypopigmentation and notching was recorded during follow up period.

## RESULTS

A total of 68 lids of 54 patients were treated. Forty four percent of the patients were males. The average age of the study group was 53year. The total follow-up period was 8 months. All patients had had previously at least one of the different treatment modalities. Twenty three patients (42.6%) were treated previously with simple mechanical epilation. The others were treated by electrolysis (15 patients), lid surgery (9 patients), and cryotherapy (7 patients). The most common known etiologic factor found among subjects was chronic blepheritis (16 patients). Other causes included trachoma (5 patients), chemical burns (3 patients) and Herpes Zoster Ophthalmicus (2 patients). Twenty eight patients (51.9%) had no known cause for their trichiasis.

The initial success rate for the whole study group following one treatment session was 61.1%. The total success rate with up to 2 additional treatment sessions within the specified period of follow up was 85.2%.

Table 1 shows recurrence rate in relation to aetiology where it was highest among patients with chemical burn and herpes zoster ophthalmicus. As regards previous treatment, patients who had previous lid surgery or cryotherapy had the highest recurrence rate as shown in Table 2.

**Table 1:** Recurrence rate in relation to etiology

Etiology	Number of patients with and without recurrence			Recurrence rate	
	Yes	No	Total		

Blepharitis	6	10	16	6/16	(37.5 %)
Trachoma	4	1	5	4/5	(80%)
Chemical burn	3	0	3	3/3	(100%)
Herpes zoster ophthalmicus	2	0	2	2/2	(100%)
Idiopathic	6	22	28	6/28	(21.4%)
Total	21	33	54	21/54	(38.9 %)

**Table 2:** Recurrence rate in relation to type of previous treatment

Type of previous treatment	Number of patients with and without recurrence			Recurrence rate	
	Yes	No	Total		

Simple mechanical epilation	5	18	23	5/23	(21.8 %)
Electrolysis	3	12	15	3/15	(20 %)
Lid surgery	7	2	9	7/9	(77.8 %)
Cryosurgery	6	1	7	6	(85.7 %)
Total	21	33	54	21/54	(38.9%)

Complications of treatment were noticed in 14 out of 68 lids (20.6%). Mild hypopigmentation was noticed in 8 lids and lid notching in 6 lids. These side effects were noticed to be more among patients treated previously with cryotherapy or lid surgery as 43% were treated previously with cryotherapy and 29% were treated previously with lid surgery. No specific correlation was found between the aetiology and the development of side effects. No other complications were noticed.

## DISCUSSION

Trichiasis is a potentially blinding condition by its continuous corneal rubbing and the resulting corneal scarring. Several conditions are known to cause trichiasis, including chronic blepheritis, trachoma, cicatricial eye inflammations, chemical burns and trauma, although a significant number of patients have no obvious cause (6, 7).

Several treatment modalities have been used to remove the trichiatic eyelashes. Each method has its own indications, success rate and complications. Epilation is the simplest method used but it is not suitable for a large number of lashes and the lashes tend to regrow within few weeks. Electrolysis is a time consuming procedure and suitable only for isolated eye lashes with a high recurrence rate. Cryotherapy, although it is successful in up to 90% of cases, but is has a high rate of complications (2-4). Argon laser is a modality of treatment for trichiasis in different centres with variable parameters. All authors reported reasonable success rates with no serious complications (8-14).

Although trichiasis is a common encountered problem in clinical practice in the Middle East area especially that related to trachoma, to the best of the investigator knowledge, few reports came from the surrounding countries on argon laser treatment for trichiasis (11, 13).

In our prospective study, we studied the results of argon laser thermoablation in a large patient series (54 patients) over a follow up period of 8 months. We managed to get an initial success rate of 61.6% after one treatment session. After a maximum of three treatment sessions, the success rate increased to 85.2%. These figures are comparable to previous reports by Campbell (15) and Huneke (16). Campbell (15) reported an 80% success rate with up to three treatments on 15 eyelids. Huneke (16) reported a 62% initial success rate in the treatment of 77 patients with trichiasis. His settings included a 50 μm spot size, 0.3 W of power and duration of 0.5 sec.

Success rate in our study is less than reported by Awan (17) where he reported results in a series of 11 patients in which he used laser beam variables: 50-200 μm spot size, exposure time of 0.2 sec, and a power setting of 1.0-1.2 W. He reported 45% success rate with one treatment, an 82% rate with two treatments and 100% success rate with three treatments. However, this remains a small series of patients. Our success rate is lower than reported by Gossman and coworkers (18) who managed to achieve an initial success rate of 88% after one treatment session and a rate of 100% after total two treatment sessions. They used higher laser settings than usual: 100 μm spot size, 0.5 sec duration, and 1.5 W power. However, they reported a 13% complication rate which is higher than other reports.

The complication rate in our series was higher than previously reported rates (15, 16, 18) where complication rates varied from nil to 13%. In ours, it was about 20.6% (14 lids). Forty three percent of the patients who had complications were previously treated with cryotherapy. This might explain the high complication rate; since previous cryotherapy might have incited some tissue damage which was later augmented and revealed by the application of laser. In addition, although we used reasonable laser settings, the amount of energy delivered might have been augmented in the more pigmented patients whom are the type of patients we encounter usually in our region. In addition, hypopigmentation is more obvious clinically in darkly pigmented patients.

In conclusion, Argon laser treatment of trichiasis is well-tolerated and easy to deliver. We recommend to implement this modality of treatment in our practice on the appropriate patients, and to continue studying the safety and effectiveness of this treatment method on specific trichiatic disorders using different laser settings. We believe this modality should replace cryotherapy in selected patients to avoid high complication rate associated with cryotherapy taking into consideration that our population are highly pigmented. It is worth trying this modality before cryotherapy since previous cryotherapy reduces the success rate of this simple safe procedure.
